# The Folk Concept of Nursing in Australia: A Decolonising Conceptual Analysis

**DOI:** 10.1111/nup.70012

**Published:** 2025-01-29

**Authors:** Jacinta Mackay, Jordan Lee‐Tory, Kylie Smith, Luke Molloy, Kathleen Clapham

**Affiliations:** ^1^ Ngarruwan Ngadju University of Wollongong Wollongong Australia; ^2^ Department of Philosophy University of Sydney Sydney Australia; ^3^ Centre of Health History Emory University Atlanta Georgia USA; ^4^ School of Nursing University of Wollongong Wollongong Australia

**Keywords:** Aboriginal and Torres Strait Islander, conceptual analysis, cultural safety, decolonisation, nursing, philosophy

## Abstract

This article presents a conceptual analysis of the contemporary understanding of NURSING in Australia and proposes strategies for decolonisation. Through historical reflection and the lens of cultural safety and critical race theory, it examines some conditions which make up this concept, including “Florence Nightingale‐influenced practices,” “intellectual practitioners,” and “whiteness in nursing.” This analysis aims to identify conditions which we take to be necessary for the folk concept of NURSING to be satisfied and which result in negative outcomes. The article explores why these conditions are plausibly included in this concept and possible objections to their inclusion. These conditions, and subsequently the concept of NURSING, are then critiqued. In this conceptual analysis of NURSING in Australia, we explore three conditions. By critically examining these conditions through the lens of cultural safety and employing decolonising methodologies, the article sheds light on the complex interplay of historical legacies, contemporary practices and potential negative outcomes within the nursing profession. The conclusions drawn propose a shift toward decolonisation, advocating for a cultural safety framework to address historical injustices and highlights possible ways in which one might amend the concept of nursing to be more inclusive of Aboriginal and Torres Strait Islander people. The need for this change is emphasised by the acknowledgement of historical conditions that perpetuated racism and hindered equitable healthcare. Ultimately, the article advocates for a comprehensive decolonisation of the concept of NURSING in Australia, urging the nursing profession to implement cultural safety for the overall well‐being of Aboriginal and Torres Strait Islander people. The authors of this article would like to acknowledge the people of the Dharawal and Dharug language group, who are the custodians of the unceded land we have worked on throughout this project. We would also like to acknowledge Aboriginal and Torres Strait Island people nationwide and warn them that some traumatic aspects of Aboriginal and Torres Strait Islander history are mentioned throughout this article. Always was, always will be, Aboriginal land. Two authors on this article identify as Aboriginal, while three do not. Two authors are registered nurses, one is an anthropologist, one is a philosopher and one is a historian.

## 1 Introduction

This article will conduct a conceptual analysis and subsequently propose ways in which we (the authors of this article) might begin to decolonise the current folk concept of nursing in Australia, henceforth NURSING.^1^ This process will focus on analysing NURSING through historical reflection and the practice of cultural safety. We begin by conducting a conceptual analysis of NURSING and proposing some conditions which we take to be part of this concept. We then justify why each condition is included in the concept and set out our specific objections to their inclusion. The chosen conditions that will be analysed are “Florence Nightingale‐influenced practices,” “Intellectual practitioners,” and “Whiteness in nursing.” A historical view will highlight the issues surrounding the current concept of NURSING in Australia, and a decolonising discussion will critique how the concept could be changed by implementing new practices such as cultural safety. Finally, a discussion about implementing this philosophy into practice will be explored, engaging a view of history informed by critical race theory.

In the article, we are concerned with the concept, NURSING. While there might be a variety of particular concepts in the vicinity to the one we will focus on, we will take NURSING to be a *folk concept*. By this, we mean a concept held more widely by people and not, for example, a niche concept that a philosopher might outline after a detailed conceptual analysis. Furthermore, we want to differentiate the folk concept *of NURSING* used by those *within* and outside the healthcare system.^2^ We refer to the former here as the *professional folk concept*, and the latter the *public folk concept*. We might expect these concepts to be similar in various ways, but we will not assume that they will be identical. In this article we are primarily concerned with the analysis of some of the features of the concept of NURSING related to the ways in which the conditions required to satisfy this concept affect culturally safe practises, inclusivity, equitable health outcomes, and the overall well‐being of Aboriginal and Torres Strait Islander people. While it seems that the use of the professional folk concept is perhaps more closely related to these effects, it is plausible that public folk concepts might also have their own consequences and perhaps different ones (especially if there are significant differences between the professional and folk concepts). However, this article will focus specifically on the *professional folk concept* and seek to identify the impacts of some of its features.^3^ Furthermore, because our analysis seeks to identify the features of this concept that have consequences on the current interactions people have within the Australian healthcare system, the target of this conceptual analysis will specifically be the concept used *currently in the Australian healthcare system*. Thus, what this article seeks to analyse is the current professional folk concept of NURSING in Australia.^4^

## 2 Methods

The concepts that we use in our day‐to‐day activities have real consequences thus it can be useful to critique the concepts that we use. The aim of this article is to provide a critique of NURSING. Our goal is not to provide a *complete* conceptual analysis of NURSING. Rather, we will focus on some particular conditions which we think make up this concept (See Jackson (1998) for a more detailed discussion regarding folk concepts and conceptual analysis). If one's concept of NURSING contains specific conditions that are in some way unjust or inequitable, it is easy to see how this might result in negative outcomes concerning things like, but not limited to; how nurses view themselves, their roles and responsibilities, nursing hiring practices and the care of their patients.

The practice of conceptual analysis, and more broadly the analysis of everyday folk concepts is widely discussed across a broad range of areas. There are many examples from different areas of the literature of this kind of conceptual analysis project that we will but undertaking. See for instance Haslanger's conceptual analysis of the folk concept of race and gender (Haslanger 2000, 2005, 2012). Another example is the wide‐ranging philosophical debates surrounding the concept of person, particularly on the nature of what kinds of changes persons can undergo such that they survive (Eklund 2002; Johnston 1989; Parfit 1984; Sider 2001). Another example is seen in the conceptual analysis of the concept of time (Baron and Miller 2015), and the empirical study of the use of such concepts in the explanations of time biases (Hodroj et al. 2022; Latham and Miller 2022; Latham et al. 2021a, 2021b). These are but a small sample of different areas in which there is discussion, analysis and sometimes critique of the particular folk concepts of which our conceptual analysis of the concept of NURSING will roughly follow stead.

To illustrate why this kind of conceptual analysis of NURSING is important and can have real world impacts we can imagine a simple example—of which clearly would have negative consequences if it were the way people actually understood the concept NURSING. Say NURSING is such that it is only satisfied by people from one ethnic background. This is to say that part of what this concept is, is that one could only satisfy it if they were from this one particular ethnic background. When I deploy this concept, for instance, when I ask to see the nurse in a hospital or imagine what a nurse looks like, I am asking and imagining a person from this one ethnic background. If this were the case it is plausible that a majority of nursing applicants and practitioners would be people with this background. A consequence of the nursing profession being practised only by people from one ethnic background could be that this leads to a lack of understanding or attention being paid to the unique and specific needs of the people they care for who are from diverse backgrounds. Thus, it is important understand these concepts that people use and analyse them for features which might result in negative consequences. If the concept or some component of it does result in such consequences, we can then reflect on possible ways that these might be reduced or eliminated.

Before we can go about analysing this concept we should be clear about how concepts themselves are structured. This is important for understanding precisely what we mean when we talk about the *conditions* upon which a concept is satisfied. Very roughly we take concepts to be the building blocks of thoughts. Here we will understand concepts as *mental representations* although nothing we will say in our analysis will hang specifically on this interpretation.^5^ Thus, we take concepts to be psychological entities located in people's heads. The theory of what concepts are and how they are structured, which we will outline below, is a general theory. Nothing we say here about what concepts are is specific to the particular concepts that we are interested in exploring (the current folk concept of NURSING in Australia).

**Figure 1:**
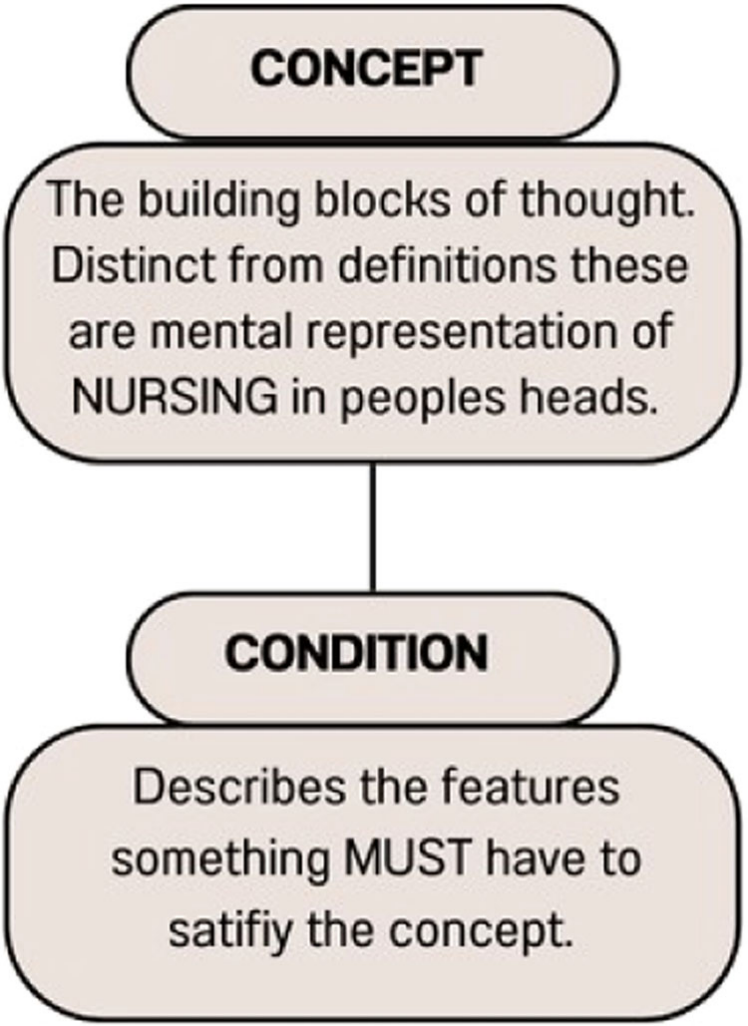
Concepts and conditions–descriptor/definitions of concepts and conditions and their relation.

Here we might look to a common example of the concept BACHELOR. Say I have the belief that *my brother is a bachelor*. This belief is constituted by some mental representations about my brother and about him being a bachelor.^6^ Assuming that there are no anomalies or extenuating circumstances one of these mental representations is the concept BACHELOR. In this article, we assume that there are such things as *shared concepts*, and subsequently, this concept is the same one which is a constituent of my mother's belief that *one of her son's is a bachelor*. Likewise, if there were a patient in a hospital or a politician deciding on health funding, and they were to have the thought; *we really need more nurses*. These thoughts presumably involve the mental representation of the same concept, NURSING. This idea is demonstrated in Figure 1.

We also take concepts to have structure. For our purposes, we will stick to a classical theory in which concepts are constituted by other more basic concepts and have a definitional structure. Again, take the example of the concept BACHELOR. This concept is seemingly composed of the more basic concepts UNMARRIED and MAN. Furthermore, this concept *is satisfied* if and only if some necessary and sufficient conditions are met. For instance, something satisfies the concept BACHELOR, if and only if it satisfies both component concepts UNMARRIED and MAN. These concepts are satisfied by something if and only if that thing meets some necessary and jointly sufficient conditions, in this case being both a man and unmarried. So then if I were to have the thought; *I should seat all of the bachelor's on the same table*, part of what makes up this thought is the concept BACHELOR. The condition upon which this concept is satisfied are such that only those people which are both unmarried and men are picked out. Thus, the thought I am having here is about my intent to seat all those who meet these conditions at the same table.

The classical view of concepts as having definitional structure has its fair share of difficulties. It has been challenged on empirical grounds (Murphy 2002; Smith and Medin 1981). It also has difficulties, at least as articulated above, in some problematic cases. For example, we might think there are edge cases in which some objects might intuitively fall under a particular concept without exactly meeting the condition outlined in its definitional structure. For example, we could imagine something that looked very much like a dog, had four legs, was fluffy, had a particular similarity in its DNA, and chased tennis balls. But it turns out that it, in fact, evolved on a different planet and thus didn't share a common ancestor with of things that we most often take to satisfy the concept DOG. It might still seem plausible that this thing satisfies the concept DOG, even if, under reflection, we would think that our concept DOG should include a condition regarding a shared common ancestor. Or perhaps we might be inclined to think that this thing does satisfy our concept DOG but just to a lesser extent then, for instance, the things which satisfy these conditions *and* share a common ancestor.

Exactly what to say about the structure of our concepts and whether these conditions should be necessary and jointly sufficient or instead are related to our concepts in some other manner is important. This is significant for our conceptual analysis of NURSING, specifically for exactly what conditions we take to satisfy the concept and how we might justify their inclusion. For instance, we earlier gave the toy example of a case in which NURSING is satisfied only by those from one ethnic background. Were we to propose this as a *necessary* condition for NURSING to be satisfied, this would clearly be open to the counterexample that there are, in fact, people from different cultural backgrounds which we think do satisfy this concept. This is why, in this article, we will instead propose higher‐level conditions which are more general in nature. For example, one might modify the previous example where instead of identifying the necessary condition of being from one ethnic background, we instead identify a more general condition. For instance, perhaps, there is a particular feature that is most prominent in those from one ethnic background such that the majority, but not all, people who satisfy this concept are those from this background.

There are other more refined accounts of concepts that have been proposed. Some examples of alternative accounts are the *prototype theory* and *theory theory* (Carey 1985, 2009; Hampton 2006). Such disputes regarding the nature and structure of concepts are beyond the scope of this article. Our analysis of NURSING will be general in nature. The goal of identifying some conditions which might result in negative outcomes will not be impeded by us taking this classical theory of concepts, so long as this analysis remains at a sufficiently high level as to avoid these kinds of fine‐grained issues and counterexamples. Relatedly it is important to note the scope and limitations of our analysis, we do not propose anything like a *complete* conceptual analysis of NURSING. We are concerned with a limited number of conditions that make up this concept. Each of these conditions will be motivated as to why we should think they are plausible parts of this concept. This will include a discussion relating to how these conditions are suitably broad as to not face any obvious problems related to them being too strong to be considered necessary for the concept to be satisfied.

In this article, we take it to be the case that one can identify these necessary and sufficient conditions of NURSING by examining our shared intuitions and examples in which it is plausible that the concept is satisfied. For each of the three conditions that we propose we will give justification for their inclusion through an appeal to; our intuitions regarding some thought experiments, an analysis of the causal‐historic practices which the nursing profession was developed from, specific examples of the concepts use in media content, and the analysis of published health policies.

## 3 Conditions

As we understand them, concepts are mind‐dependent entities, located in people's heads.^7^ Concepts are closely related to linguistic definitions but where linguistic definitions concern the meaning of words, concepts concern the meaning of thoughts (Isaac, Koch and Nefdt 2022). We are in the business of conducting a *conceptual analysis* of NURSING. That is, we seek to understand some of the features of this concept and we are not going to engage in any kind of detailed prescriptive work regarding suggesting or arguing for the modification of this concept. That is, our task is a descriptive one; we want to describe NURSING that people have and use, specifically, the conditions that make up NURSING which determine what things satisfy this concept. The result of this work naturally leads into this latter project of *conceptual engineering* and we will suggest some work that needs to be undertaken in this regard, proposing potential decolonising ways forward, but our primary focus is on deploying historical reflection and cultural safety to undertake the conceptual analysis of NURSING.^7,8^ Exactly how concepts relate to these specific conditions and in what sense we take things to satisfy concepts will be unpacked further below. Furthermore, the “conceptual analysis” that exists in the field of nursing philosophy and literature to date is wholly different from this approach, as we take a strong philosophical approach to the term concept as described above, rather than a descriptive approach as seen in other literature (Hellman 2024).

In this section, we focus on three specific conditions of NURSING which have roots in historical colonising practices, which we argue can result in negative outcomes in present‐day healthcare settings. Notably, we are not suggesting that these three conditions are the only conditions that are required to be a nurse but suggesting that they are part of what is required. That is, we take these to be necessary but not sufficient conditions for this concept to be satisfied. Here we employ a decolonising methodology and critical race theory to understand what it takes for some conditions to have negative outcomes. A decolonising methodology has been designed as a response to the history of the colonisation of Australia and more broadly the world, and it acknowledges the colonisation of every area of research. It is a methodology that identifies the colonisation of all things and works towards decolonising through active thought and movement away from the colonising actions of the past and colonial mentalities (Paradies 2016; Smith 2012). Critical race theory identifies colonising and racist underpinnings in organisations, institutions and histories (Delgado, Stefancic, and Harris 2017). Literature supports the use of critical race theory in analysing health disparities, health systems and historical sources and highlights that these systems are inherently racist to those who do not fit the shifting criteria of “whiteness” (Adebayo et al. 2022; Zaidi et al. 2023). Furthermore, critical race theory is suggested as a way to identify colonisation and actively decolonise practices and institutions (Gatwiri, Rotumah, and Rix 2021). We will use both decolonising methodologies and critical race theory to ensure that our conceptual analysis views these conditions from a critical decolonising point of view and aims to decolonise the conditions to work towards a decolonised concept.

Finally, cultural safety was first coined by Ramsden (2002) in the context of the New Zealand healthcare setting and First Nations people. In Australia, it is defined by the Congress of Aboriginal and Torres Strait Islander Nurses and Midwives (CATSINaM) as “an outcome of nursing and midwifery education that enables safe service to be defined by those that receive the service and is a way of providing care that is holistic, free of bias and racism, challenges belief based upon assumption and is done in a respectful manner.” (CATSINaM 2022b). Notably it is also defined by the person receiving care rather than the caregiver (CATSINaM 2022b). Cultural safety is a prevailing theme throughout this article and will highlight the importance of historical analysis within our conceptual analysis.

## 4 Conceptual Analysis of Nursing

**Figure 2:**
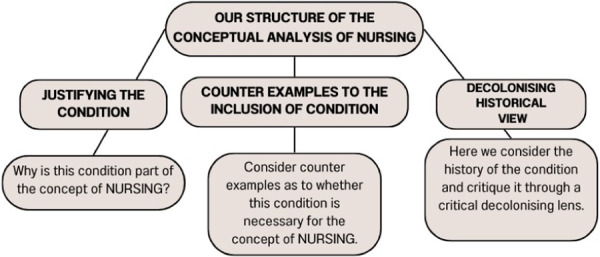
Analysis structure–our conceptual analysis structure within the article.

This thought exercise will highlight some of the simpler constitutive concepts that form the conditions upon whether something satisfies this concept. In the real world, NURSING is clearly a complex concept built upon a large number of other concepts. In this article, we limit our focus to three conditions of which we take to plausibly result in negative outcomes. For each condition we introduce, we will explain its significance, justify its inclusion, outline some possible objections to it being a necessary condition for NURSING to be satisfied, and offer a critique of it. The three conditions which we focus on are; *Florence Nightingale‐Influenced Practices*, *Intellectual Practitioners* and *Whiteness in Nursing*. This analysis will allow a preliminary examination of what makes up NURSING that can result in negative outcomes. Through this analysis we will suggest ways in which one might go about the process of decolonising NURSING. Through the lens of critical race theory, we will examine each of these necessary conditions and, in turn, highlight colonisation, whiteness, inequalities and racism, both historic and current. Critical race theory provides us with a view of these conditions that highlights the need for decolonisation, while cultural safety will make the basis of our recommended changes to decolonise. Importantly, the authors would like to note here that this is a thought exercise and that a broad Community consultation would be the ideal way to ultimately identify the impacts, and achieve the successful decolonisation, of NURSING. This structured approach has been simplified in Figure 2.

## 5 Florence Nightingale‐Influenced Practices

Many nursing practices today have their roots in Nightingale's teachings. For clarity in this article, we will focus on only one of the aspects of nursing she pioneered the teaching of, particularly cleanliness/sanitation or more modernly, infection control (Loveday 2020; Nelson and Rafferty 2010). Infection control is a fundamental part of nursing care and/or knowledge today, and although Nightingale did not know it as it is today, she undoubtedly contributed to its current state. Nightingale also pioneered modern forms of nursing schools and teaching programs which taught nurses knowledge of what we now know as infection control (Gilbert 2020). Although she was not the creator of these ideas, she heavily influenced the teaching of nurses and insisted on its compulsory inclusion in education and practice (Gilbert 2020; Nelson and Rafferty 2010). Her model of teaching and ward design was adopted widely in the English speaking world (Nelson and Rafferty 2010). Thus, we propose that there is a condition of having knowledge of these practices that is necessary for NURSING in Australia to be satisfied. If someone fails to follow these, for instance, they fail to have knowledge of infection control, they subsequently fail to satisfy NURSING. This condition requires that nurses have knowledge of the modern skill of infection control in relation to patient care, but not necessarily that they are aware of Nightingales' link to it.

To justify that this condition is part of NURSING we look to health policy and the influence Nightingale still has over the profession and education. Nightingale receives recognition as the mother of nursing, the lady with the lamp and the foundations of nursing in Australia came from her and her training schools (Karimi and Masoudi Alavi 2015). She created the modern nursing school in the United Kingdom and, when serving in the British army, reformed the practice of infection control and nurse training (Karimi and Masoudi Alavi 2015; Nightingale and McDonald 2001). Furthermore, in Australia, specifically, our first nursing school was established by Lucy Osbourne under the instructions of Nightingale (Godden 2006). Students trained by her would become the first Nightingale nurses in Australia and successfully spread her teaching throughout the Australian nursing education and hospital system (Godden 2006). Due to the colonisation of Australia and the influence that British culture has had on our society's development, Nightingale has also influenced hospitals and training schools in Australia due to her large influence in the United Kingdom. Health policy solidifies the continued use of infection control within modern nursing practice and education (NSW Health 2014, 2023; Schwartz 2022). Nightingale's influence is also felt with the International Day of the Nurse is held on Nightingale's birthday, her work is a core element in the foundations of nursing taught to Australian undergraduate nursing students, Florence Nightingale Medals are awarded to outstanding Australian nurses, and there is a large amount of literature about her and her work in relation to Australian nursing (Broodkoorn 2020; Karimi and Masoudi Alavi 2015; Nightingale and McDonald 2001; Schwartz 2022; Wang, Zhu and Duan 2021). This highlights that Nightingale, to this day, has a major impact on the way we view nurses and the way we expect nurses to act and practice. Furthermore, her ideals have been ingrained into nursing knowledge and practice today (Best 2015; Karimi and Masoudi Alavi 2015; Nightingale and McDonald 2001; Stake‐Doucet 2020; Wang, Zhu and Duan 2021). This results in NURSING being the way that it is because of the influence and development of the profession that Nightingale had.

Some may object to this condition being necessary to be a nurse in Australia, we hypothesise objections may include, nurses do not have knowledge of or practice modern infection control the same way Nightingale did. In response to this we point out that although nursing practices have advanced, the fundamentals stay the same. Literature also supports Nightingales pioneering work in the infection control field, for example her early adoption of hand washing practices or the relevance of her environmental hygiene theory in COVID‐19 infection control (Gilbert 2020; Kolagari 2023; Martini and Lippi 2021; Olans et al. 2022). While she may have opposed germ theory and practised miasma, our condition centres not on her scientific rigour but on her continued influences in practice today (Aravind and Chung 2010; McEnroe 2020). Furthermore, her belief in miasma is also defended in literature stating how this led to beneficial reform for patients (Aravind and Chung 2010; McEnroe 2020). As we have clarified above, her influence highlights her veneration within Australian nursing and the literature supporting her use of miasma when viewed with a critical lens only strengthens our stance on her influence today despite her flaws. There are many objections to why some of Nightingale's ideas and teachings are not an accurate picture of NURSING in Australia today. Her views on women in nursing, colonisation, race and religious values are all areas that today do not accurately depict an Australian registered nurse (Godden 2006; Karimi and Masoudi Alavi 2015; Loveday 2020; Nightingale 2021; Nightingale and McDonald 2001). However, when focusing on the specific areas noted within this condition her foundations of practice are still found to be vital to nursing knowledge and practice today and therefore are included in the concept (Olans et al. 2022).

Historically, Nightingale's writings depict her view on Indigenous lives across the commonwealth and her objection to their “Barbarism” (Stake‐Doucet 2020). She also speaks of how important cleanliness is to nursing practice but does not regard Indigenous peoples as clean (Stake‐Doucet 2020). While nurses today may not hold this same belief, a critical race view highlights that elements of Nightingale's ideas come from her flawed nineteenth‐century racist worldview and are generally upheld by the white norm (further explored in our condition of whiteness). This brings into question if NURSING today is satisfied with its racist history that pays homage to women who contributed to the colonisation of thousands of Indigenous lives or if it needs changing? To continue this critique of the condition centred around Florence Nightingale and her teachings, the concept required that a nurse knowingly or unknowingly base selected practices on her teaching as the ‘mother of modern nursing’ (Karimi and Masoudi Alavi 2015). However, in a decolonising process, we propose that Nightingales' influences be separated from the concept and that her undertones of racism be taken with her as it will be difficult to have a truly culturally safe space with her ideals remaining. The CATSINaM (2017) highlights the importance of teaching history to nurses; we suggest Nightingale is still included but with a truth telling and critical race theory lens allowing for critical historical reflection on her impact. As discussed above her ideals relating to cleanliness and fresh air within the healthcare setting revolutionised the care of people in the 19th century, however modern nurses have largely outgrown the ideals that exclude people of colour from care. A decolonised and culturally safe nursing would maintain some of the practices Nightingale pioneered; however, it removes her lingering political and social views from the condition. Although resistance to change may be felt throughout the process, the fundamentals of practice are unlikely to drastically change, rather the motivations behind the practices, emphasising the inclusion of people of colour and Aboriginal and Torres Strait Islander people. The current condition of Nightingales fundamental practices is not inclusive to Aboriginal and Torres Strait Islander people and a decolonising process is needed.

## 6 Intellectual Practitioners

Intellectual modern nurses utilise their skills in critical academic thinking, emotional intelligence and are tertiary‐educated (Bulmer Smith, Profetto‐McGrath and Cummings 2009; Lusk et al. 2001). The idea that nurses are intelligent, educated and experienced individuals who use their knowledge and skills to inform their clinical judgement is of fundamental importance for nurses (NMBA 2016). Thus, we propose that NURSING includes the condition that one must be an intellectual practitioner, intellectual in our case meaning someone who utilises their skills in critical thinking, emotional intelligence and is tertiary‐educated (Bulmer Smith, Profetto‐McGrath and Cummings 2009; Lusk et al. 2001). For instance, were someone to fail to be educated they would fail to satisfy NURSING.

This condition has been included as nursing has become an intellectual profession, with registered nurses requiring a university degree to enter the profession (Department of Health and Aged Care 2023). We also see an increasing rate of registered nurses undertaking and being encouraged to undertake postgraduate study in Australia to specialise their practices, particularly in speciality areas such as the Intensive Care Unit; some areas require 50% of staff to hold these qualifications (Chamberlain, Pollock and Fulbrook 2018). While postgraduate qualifications are not required in this condition, they do emphasise the move towards an increasing intellectual NURSING. Nurses' critical thinking skills and emotional intelligence is widely supported by the literature surrounding their importance in being a competent nurse (Bulmer Smith, Profetto‐McGrath and Cummings 2009; Zuriguel Pérez et al. 2014). Furthermore, the registered nurse standards of practice for Australia highlights the necessity of critical thinking, utilising available evidence and research (NMBA 2016). These standards must be upheld to maintain a nursing registration. Similarly, continuing professional development and recency of practice must be maintained, highlighting the importance of continual learning, maintaining intellectual nursing practice (NMBA 2022). Therefore, under the current concept of NURSING in Australia, nurses are required to be intellectual thinkers and practitioners.

A possible objection to the inclusion of the condition of intellectual practitioners within the concept is that there are some nurses who are hospital‐trained and have not attained a university degree. We counter this by highlighting the definition of tertiary education and that hospital‐trained nurses are still tertiary‐educated, experienced professionals and meet the condition. As stated above these nurses also maintain their nursing registrations which requires the same professional development and critical thinking (Australian Nursing and Midwifery Accreditation Council, A 2019). Again, this reinforces that all nurses within the Australian setting are intellectual practitioners.

A critique of this condition is that it disregards Aboriginal and Torres Strait Islander knowledge and ways of practising health care. This is due to nursing being a biomedical model and valuing biomedical outcomes whereas a comprehensive Aboriginal and Torres Strait Islander healthcare model values different aspects of care (Pearson et al. 2020). The knowledge of Aboriginal and Torres Strait Islander health practitioners is not valued within the tertiary based system as it is not a recognised university degree and does not count as credit toward a nursing degree which is required to become a registered nurse. Furthermore, although the accreditation council stated pre‐registration nursing students must be taught about Aboriginal and Torres Strait Islander health, there is no room for a recognition of prior learning for Aboriginal ways of knowing being and doing (Council 2019; Wilson 2008). For example, if someone was recognised in their Community for their knowledge of health and delivered healthcare to the Community for years, this would not be recognised if they decided to enrol in a nursing degree, they would have to complete subjects in Aboriginal and Torres Strait Islander health but these are almost always focused on a deficit model or health as a “problem” in these Communities, not from the point of view of understanding Aboriginal and Torres Strait Islander health knowledge or practices. Aboriginal and Torres Strait Islander healthcare practitioners are therefore not included in this concept, and are arguably not given the same opportunity to be included. There are also many barriers to university‐based nursing education for Aboriginal and Torres Strait Islander people historically and currently (Doran, Wrigley and Lewis 2019; Henry, Houston and Mooney 2004; Povey et al. 2023). The *Bringing them Home Report* shared firsthand accounts of many Aboriginal and/or Torres Strait Islander women who wanted to be nurses but were taken to stations to work as domestics instead or blatantly told they could not be nurses due to their race (National Library of Australia 1998–2002). History also shows that those who did become nurses faced more barriers than their peers (Best and Gorman 2016). Today reports consistently find that Aboriginal and Torres Strait Islander people are not at parity to non‐Indigenous people for higher education and that the higher education setting is institutionally racist and harmful to them (Education 2015; Povey et al. 2023). These barriers result in less Aboriginal and Torres Strait Islander people meeting the condition to satisfy NURSING in Australia and Aboriginal and Torres Strait Islander knowledge not being recognised within the current concept.

To continue the critique of this condition it needs to account for cultural health practices and practitioners and also ensure that Aboriginal and Torres Strait Islander people have equitable access to the university education requirements for becoming a registered nurse (Hassmiller and Wakefield 2022). The university‐based system is historically and currently a colonising force within Australia, emphasising western privileges and the biomedical model (Wilson 2008). It also does not account for Aboriginal and/or Torres Strait Islander ways of knowing, being and doing (Wilson 2008). This results in healthcare education and delivery being from a colonised perspective. To counteract this, education must be given in culturally safe ways and resources made with a large Community consultation. Furthermore, intellectual value must be placed on the knowledge of Aboriginal and Torres Strait Islander people, allowing for oral histories and cultural practices to be a vital part of intellectual training and education. The barriers discussed above to Aboriginal and Torres Strait Islander students need to be addressed and counteracted to allow for equitable access to education settings and therefore entry into the nursing profession (Education 2015; Povey et al. 2023). Finally, it is forecast that even higher levels of education will be required of nurses in the future, this condition needs to account for that and ensure that Aboriginal and Torres Strait Islander people are a part of this education system to provide decolonised, culturally safe practice (Hassmiller and Wakefield 2022).

## 7 Whiteness

Whiteness plays a major role in nursing in Australia. Whiteness is the dominance of white culture both structurally and systematically (Nielsen, Alice Stuart and Gorman 2014). The theory of whiteness suggests that the majority of both academic and nursing staff fit the “white norm” and have a blindness to the needs of First Nation's people (Sullivan 2006). This theory suggests that it is not done with maleficence, but rather, there is no active thought to include those who do not fit into society's white norms (Sullivan 2006). This theory of whiteness has ensured that all nurses in Australia are competent in providing care to the white norm but may not be aware of what Aboriginal and Torres Strait Islander people require. Therefore, we propose that a condition of NURSING is that nurses are competent in providing care to the white norm of Australia. That is, were someone not competent in providing specific care to people who match the white norm, regardless if they were competent in providing other aspects of care, or care to those who do not match this norm they would not satisfy NURSING.

The theory of whiteness has been included as a condition of NURSING in Australia due to the literature surrounding the theory suggesting it is a major factor in how nurses deliver care (Nielsen, Alice Stuart and Gorman 2014; Povey et al. 2023). With such a large number of people in Australia fitting the white norm, the theory of whiteness highlights how this can affect nursing education and practice (Sullivan 2006). This is also evident broadly in healthcare education today, with examples such as an under representation of darker skin tones in dermatological education resources (Babool et al. 2022). These factors result in nursing education and practice reinforcing the white norm.

Objections to the condition that all nurses are aware of the needs of the white norm due to the whiteness within the nursing profession, include that not all nurses meet the white norm themselves. However, our condition clearly states that nurses do not need to fit the white norm, they only need to know how to care for those who do. As literature supports that the white norm makes up the majority of people receiving care within the Australia nursing setting, nurses are only required to know how to appropriately care for this norm (Nielsen, Alice Stuart and Gorman 2014; Sullivan 2006). Another objection may be that many nurses are aware of how to care for Aboriginal and Torres Strait Islander people, however we argue that while some may, not all do. Furthermore, there is further research that needs to be conducted regarding the comparison of whether nurses feel they meet care needs compared to whether Communities feel care needs are met.

Historically, there are many examples of this whiteness playing out to the detriment of Aboriginal and Torres Strait Islander people. In the *Bringing Them Home* report, many stolen children report wanting to be nurses but being told they were not able to do so due to their skin colour and culture (National Library of Australia 1998–2002). Similarly, there are many accounts of nurses playing key roles in the segregation and assimilation of Aboriginal and Torres Strait Islander peoples within hospital settings, this includes the taking of Aboriginal children, the forced sterilisation of women and the segregation of patients and nurses within these settings (Best and Gorman 2016; National Library of Australia 1998–2002). These accounts are extreme examples of the detriment the whiteness of the nursing profession can bring to those who do not fit the norm. Currently only 1.3% of nurses are Aboriginal and/or Torres Strait Islander which is below parity in Australia given that Aboriginal and Torres Strait Islander people account for 3.8% Percent of the total population (Australian Bureau of Statistics 2021; CATSINaM 2022a). Historical and current factors result in barriers to employment and care for those who do not meet the norm but also highlight how the needs of the white norm are constantly being met.

To continue the critique of the whiteness condition of NURSING, the reality of nurses appropriately caring for the white norm but not those who do not meet it, is harmful to people of colour and Australia's Aboriginal and Torres Strait Islander Communities (Gatwiri, Rotumah and Rix 2021; Nielsen, Alice Stuart and Gorman 2014; Povey et al. 2023; Sullivan 2006; Zaidi et al. 2023). Using a critical race theory lens to ensure that all aspects of nursing care and history are viewed not from the coloniser's viewpoint, emphasises the effects of whiteness within Australian healthcare settings (Nielsen, Alice Stuart and Gorman 2014). The condition hinders an equitable healthcare environment for all. Counteracting the whiteness within these settings by changing the actions and values of the majority of nurses who fit the white norm is a required change within this conceptual block to move forward and decolonise nursing. Cultural safety would result in all nurses practising cultural awareness, sensitivity and safety (Best and Fredericks 2021). That is, it would require asking their colleagues and patients what they need to feel culturally safe in healthcare settings and delivering that to them. It would also result in all nurses being aware of their own cultures, sensitive to how this is different from others and then being able to adjust to create safe spaces for all (Best 2021; Ramsden 2002). A suggested new condition, therefore, would include all nurses meeting this new culturally safe norm, which would also address the requirements under the code of conduct to do so (NMBA 2018). Creating an equitable healthcare environment for nurses and their patients would result in all nurses practising cultural safety, resulting in their colleagues and patients achieving equity within the healthcare setting.

## 8 Discussion

This thought exercise of critically analysing NURSING in Australia and the idea of decolonising through cultural safety should be the basis of a new approach to conceptualising nursing. As pointed out, in many of the current conditions of NURSING there are many fundamental aspects of modern nursing that either historically stem from or still contribute to racism within the healthcare setting (Gatwiri, Rotumah and Rix 2021; Henry, Houston and Mooney 2004). It is widely acknowledged in literature that change is needed within the nursing profession to decolonise the practice for the wellbeing of our Aboriginal and Torres Strait Islander colleagues and patients (Geia et al. 2020; Hine et al. 2023). Furthermore, a process of decolonisation can also be utilised to assist in the improved care of all those from culturally and linguistically diverse backgrounds in Australia. Statistics show that Aboriginal and Torres Strait Islander people are not entering the profession of nursing and similarly, leaving hospitals against medical advice well above parity (Australian Institute of Health and Welfare 2023; Department of Health and Aged Care 2019). As of 2019, Indigenous people in Australia had a 5.2 times higher rate of leaving hospitals against medical advice than nonindigenous patients (Australian Institute of Health and Welfare 2023). These statistics emphasise the need for change in our current concept and the potential a re‐engineering could have on our nursing workforce and patient outcomes. We hypothesise that this could be achieved through a decolonising, culturally safe process.

The acknowledgement that history and truth‐telling practices are a vital part of culturally safe nursing for Aboriginal and Torres Strait Islander people is a key factor of this article (Best and Fredericks 2021). Bringing to light the truth of the history surrounding the current concept of NURSING is a vital exercise in the process of decolonising as we cannot move forward without acknowledging the wrongs of the past. Our analysis through a critical race theory lens highlights the importance of context when analysing NURSING and the racist undertones some of our nursing practices have. This historical view also highlights how many of the fundamentals of nursing practice have gone unquestioned for much of Australian history. This brings into question whether nurses today in Australia are happy to continue practices that have historically racist fundamentals or if a change and re‐engineering of the concept is needed (or demanded) by modern nurses. We believe that a decolonising, re‐engineering process is vital for the improved care of Aboriginal and Torres Strait Islander patients and the experiences of Aboriginal and Torres Strait Islander nurses.

## 9 Conclusion

This thought exercise argued that the current concept of NURSING in Australia has a culturally unsafe past and that this translates into nursing care today, through the conditions of which we take this concept to be satisfied. A historical analysis highlighted the problematic origins of many of these practices and some potential fundamental flaws that allow for racism to translate into nursing care today. Potential objections to each condition being a required part of NURSING and critiques of conditions have been explored. The process of changing NURSING or re‐engineering the conditions would require institutional change and a process of decolonisation throughout the nursing profession. It is clear that this change to a decolonised NURSING is needed to assist Aboriginal and Torres Strait Islander nurses and patients to feel culturally safe and to safely participate in healthcare. We believe that this can be achieved by nurses embracing history and creating culturally safe environments for themselves, their colleagues and their patients.

## Conflicts of Interest

The authors declare no conflict of interests.

## Data Availability

The authors have nothing to report.

## Endnotes

1 In this article, we will follow the commonly used convention of capitalising concepts to distinguish them from the ordinary use of these same words. For instance, NURSING is capitalised when referring to the concept to avoid confusion with the use of “nursing” when referring to the practice of nursing.

2 There are various things one might say regarding what it takes for the concepts held by different people, or the same person at different times, to count as being the *same* or a *shared* concept. Much of that discussion will depend on exactly what concepts are. Later, we will subscribe to a simplified view regarding the ontology of concepts however since what we say in this article will depend little on precise details of what concepts are, and such details are not within the scope of this conceptual analysis, we will remain neutral regarding what to say about *the nature of shared concepts*. We will assume that there is *some sense* in which concepts are jointly held by the folk, or there are some features of the distinct concepts held by the folk are shared, which the idea of a *shared concept* is tracking. This minimal assumption seems plausible, not least in part because people deploy their concepts and are, for the most part, able to engage in effective communication, which we might not expect if this assumption were rejected.

3 This is not to dismiss the potential importance of identifying the potential differences between these concepts or the importance of identifying the consequences that might arise out of the features of the public folk concept, but only to clearly identify the target of the conceptual analysis of this article.

4 Henceforth we will drop the qualification of *folk concept, professional, currently*, and *in Australia*. However, this should be implied whenever we refer to the concept of NURSING in what follows.

5 The ontology of concepts is an ongoing dispute. We will not be able to unpack in detail the view of concepts as mental representations here. However, this view is relatively widespread across some disciplines. For a more detailed account and defence see Fodor (1975, 1987) and Sutton (2004). Some support of this view can be seen in the field of cognitive science, see for instance Carey (2009), Pinker (2007) One competing view is that concepts are, or are associated with some particular abilities that cognitive agents process, see for instance Dummett (1993), Bennett and Hacker (2008) and Kenny (2010). Another competing view is that concepts are abstract entities, see for instance, Peacocke (1992).

6 This is not always how the relationship between beliefs, mental representations and concepts are taken to be even across views which do take concepts to be mental representations, see for instance Fodor (1987) for a more detailed discussion.

7 For a detailed overview of the general project of conceptual engineering see Cappelen (2018).

8 Although through our identification of the potential issues that arise from our analysis of some of the cultural and historic influences that influence these conditions upon which this concept is satisfied this is a natural and worthy future project.
